# Nanocelluloses Reinforced Bio-Waterborne Polyurethane

**DOI:** 10.3390/polym13172853

**Published:** 2021-08-25

**Authors:** M. E. Victoria Hormaiztegui, Diana Marin, Piedad Gañán, Pablo Marcelo Stefani, Verónica Mucci, Mirta I. Aranguren

**Affiliations:** 1 Facultad de Ingeniería, Instituto de Investigaciones en Ciencia y Tecnología de Materiales (INTEMA), UNMDP, CONICET, Av. Juan B Justo 4302, Mar del Plata 7600, Argentina; mevhormaiztegui@frlp.utn.edu.ar (M.E.V.H.); dianacatalina86@gmail.com (D.M.); pmstefan@fi.mdp.edu.ar (P.M.S.); vmucci@fi.mdp.edu.ar (V.M.); 2 Centro de Investigación y Desarrollo en Ciencia y Tecnología de Materiales (CITEMA), Facultad Regional La Plata, Universidad Tecnológica Nacional (UTN)-Comisión de Investigaciones Científicas de la Provincia de Buenos Aires (CIC), Av. 60 y 124, Berisso 1923, Argentina; 3 Facultad de Ingeniería Química, Universidad Pontificia Bolivariana (UPB), Circular 1, No 70-01, Medellín 050031, Colombia; piedad.ganan@upb.edu.co

**Keywords:** bio-based waterborne polyurethane, castor oil, bacterial cellulose, cellulose nanocrystals

## Abstract

The aim of this work was to evaluate the influence of two kinds of bio- nano-reinforcements, cellulose nanocrystals (CNCs) and bacterial cellulose (BC), on the properties of castor oil-based waterborne polyurethane (WBPU) films. CNCs were obtained by the acidolysis of microcrystalline cellulose, while BC was produced from *Komagataeibacter medellinensis.* A WBPU/BC composite was prepared by the impregnation of a wet BC membrane and further drying, while the WBPU/CNC composite was obtained by casting. The nanoreinforcement was adequately dispersed in the polymer using any of the preparation methods, obtaining optically transparent compounds. Thermal gravimetric analysis, Fourier-transform infrared spectroscopy, field emission scanning electron microscopy, dynamical mechanical analysis, differential scanning calorimetry, contact angle, and water absorption tests were carried out to analyze the chemical, physical, and thermal properties, as well as the morphology of nanocelluloses and composites. The incorporation of nanoreinforcements into the formulation increased the storage modulus above the glass transition temperature of the polymer. The thermal stability of the BC-reinforced composites was slightly higher than that of the CNC composites. In addition, BC allowed maintaining the structural integrity of the composites films, when they were immersed in water. The results were related to the relatively high thermal stability and the particular three-dimensional interconnected reticular morphology of BC.

## 1. Introduction

The continuous growing global interest in reducing the environmental pollution has triggered and sustained the research and development of environmentally friendly polymeric materials to replace polymers of synthetic origin in different applications [1,2]. 

In particular, polyurethanes (PUs) have received much attention. They are versatile polymers that find applications in various fields in the form of elastomers, foams, matrices of structural composites, fibers, adhesives, coatings, etc. Since PUs are soluble in organic chemicals, their traditional use as coatings and their preparation as thin self-standing films are associated with the release of volatile organic compounds (VOCs) into the atmosphere. Therefore, important efforts have been devoted to reduce this problem, and thus, during the last few decades, there has been a growing preference towards the use of waterborne polyurethanes (WBPUs) that consist in the stable suspensions of PU nanodroplets in water [3]. With the introduction of bio-based polyols in the market, the preparation of bio-based WBPUs has also progressed with the aim of producing greener alternatives to traditional materials [4,5,6,7]. 

The synthesis of WBPUs requires the incorporation of an emulsifier that allows the stabilization of the PU aqueous dispersion. Frequently, this is achieved by using a short diol containing an ionizable group in its molecular structure as a co-monomer. Anionic and cationic WBPUs can be prepared, although the former are more common. In the case of an anionic WBPU, the internal emulsifier is a diol containing also a carboxyl group, while a counterion must be incorporated in aqueous solution media [8,9]. Bio-based WBPUs are also prepared with this technique, and vegetable oils have shown to be a particularly attractive source for the preparation of polyols. These polyols are obtained via the chemical modification of oil or from the preparation of monomers from which the polyols are synthesized [4,10,11]. In few cases, vegetable oil can be used directly as a polyol, which is the case of castor oil (CO) that has hydroxyl groups in esterified ricinoleic acid chains [4].

In order to improve or tailor properties to meet specific requirements, PU aqueous dispersions have also been modified by the addition of different nanoparticles, either inorganic or bio-based ones, to produce self-standing films or coating formulations [3,12,13,14,15,16]. Transparent films, mostly from anionic WBPUs, have been produced from CO-derived monomers, also containing different nanofillers/reinforcements (cellulose nanocrystals (CNCs) [17,18,19], nanosilica [13,20], nanosilver [21], and nanoclays [22]).

One of the most studied bioreinforcements is cellulose, because of its worldwide availability, outstanding properties, and low cost [23,24,25,26]. In addition to these benefits, micro- and nanocelluloses can be handled in aqueous suspension and thus easily incorporated in the bio-based WBPU formulation [19,27].

When cellulose is obtained from plants, it must be separated from the other components present in the raw materials. This top-down process consists in the disintegration of the vegetable biomass, followed by the purification of the cellulose and then usually a combination of mechanical and chemical (or enzymatic) steps that lead to the defibrillation of the cellulose microfibers, to obtain fibrils of a few nanometers in thickness. When the final product is CNCs, the process continues with the acidolysis of the fibrils (a strong acid such as sulfuric acid is frequently used) that degrades preferentially the amorphous regions and allows ending up with acicular nanoparticles, CNCs, with thicknesses usually in the range of 5–10 nm and lengths of 150–200 nm [23,25,28,29]. CNCs have aspect ratios usually above 10 and present low density, high strength, low thermal expansion, and very high specific surface areas [30]. Due to these characteristics, it has been used as an emulsifier, a filler, a stabilizer, and a rheological thickener. Some researchers have investigated the use of CNCs as a reinforcement of PUs and WBPUs, taking advantage of the interfacial interaction developed between materials through H-bonds. It has been reported that cellulose–PU interactions have effects on the phase separation of segmented PUs and on the crystallization of some of these polymers [18,31,32]. 

On the other hand, bacterial nanocellulose (BC) is obtained via a bottom-up process, which consists of the external secretion of different bacteria, most frequently *Acetobacter* and *Glucanobacter*, by that produce a nanofibrillar’s three-dimensionally (3D) entangled pellicle as a protective measure. The nanofibrils (thickness: 2–4 nm) are extruded through specific points in the bacterial cell wall and aggregate to form long ribbons of high crystallinity, but also with amorphous segments that make them very flexible [33,34]. 

After the BC pellicles are carefully washed to remove any impurity, they can be used in their hydrated state or as freeze dried membranes [35]. Compared to CNCs, BC is a purer nanocellulose, since no hemicelluloses or lignin are present. It is also highly crystalline with excellent strength and flexibility [30]. These characteristics have lead to its use in the development of biomedical materials (scafolds for hydrogels and replacement materials) [23,36,37]. Moreover, in some cases, they have been used as a source in the production of nanocrystals [38].

The introduction of *Komagataeibacter medellinensis* for producing BC is relatively recent. This strain presents the novel and very interesting characteristic of being able to grow in more acid conditions than other bacteria of more common use. This is not a minor advantage: it is possible to cultivate it using undervalued food wastes and agricultural by-products, such as rotten banana and mango and cheese whey. Its capacity of growing at low pH results in a competitive advantage over other microorganisms [39,40]. 

As in the case of CNCs, BC can also be used as a reinforcement of WBPUs. However, comparatively, a lower number of works have been reported that address the preparation of WBPUs with BC nanocomposites. For example, a recent review [41] on WBPU and PU urea dispersions addressed the combination of these polymers with nanocelluloses, including only CNCs and microfibrillated celluloses. The interfacial chemical interaction between the polymers and these plant-based nanocelluloses can differ from those of BC. While CNCs frequently contain sulfate groups on the surface and micro- or nanofibrils of cellulose may contain carboxyl groups on the surface due to the chemicals used in the process of obtention, these groups are absent on the surface of BC [23,30,41]. Urbina et al. prepared such a composite by the immersion of a wet BC membrane into a commercial synthetic WBPU [42]. The composite showed shape memory behavior and was activated by immersion in water at 40 °C. The authors found a significantly enhanced recovery because of the incorporation of BC. Feng et al. also prepared a WBPU composite with BC using a commercial synthetic polyol to prepare a nasal stent [36].

In this work, an ecofriendly WBPU based on a biopolyol (CO) has been used as well as two different types of nanocelluloses as reinforcement (CNCs and BC). Few works have been focused on the comparative behaviors of these nanocelluloses, and an even lower number has considered the use of a bio-based WBPU. The use of the strain *Komagataeibacter medellinensis* is also a novelty that introduces further interest because of the particular characteristics of these bacteria. The work discusses the different requirements of the preparation of self-standing films made from the two nanocelluloses and compares the final properties of the composites relating them to the morphologies of the nanocelluloses and their surface chemistry. The bio-based films may have uses in different areas such as protective and decorative coatings (CNC composites) and membranes and biomedical materials (BC composites).

## 2. Materials and Methods

### 2.1. Raw Materials

CO (Parafarm^®^; OH number = 177.21 mg/mg of CO; f = 2.9) and dimethylolpropionic acid (DMPA; purity: 98%; f = 2; Sigma-Aldrich Corp., St. Louis, MO, USA) were dried under vacuum before use it. Isophorone diisocyanate (IPDI; purity: 98%; NCO number = 24.06% determined by ASTM D2572; f = 2), dibutyltin dilaurate (DBTDL; purity: 95%), triethylamine (TEA; purity: 99%), acetone, and dimethylformamide (DMF) were purchased from Sigma-Aldrich Corp. and used without purification. 

In order to obtain CNCs, microcrystalline cellulose powder (MCC) purchased from Sigma-Aldrich Corp. was used. Sulfuric acid (*w*/*v*: 98%; Anedra) was used to proceed with the cellulose acid hydrolysis. Bi-distilled water and Spectra/Por Standard RC dialysis tubing were used in the dialysis of the cellulose crystals suspension. Citric acid (Biopack), glucose (Britania), peptone (Britania), sodium phosphate dibasic anhydro (Na_2_HPO_4_; Cicarelli), and potassium hydroxide (KOH; Merck) were used to obtain bacterial cellulose.

### 2.2. Synthesis of the WBPU

CO and DMPA (OH equivalent molar ratio of CO to DMPA = 1.0) were fed into a five-necked glass reactor. The reaction was carried out under a N_2_ atmosphere to reduce any contribution from ambient humidity. Then, IPDI (NCO/OH ratio = 1.4) and DBTDL (1 wt.% with respect to the total reaction mass) were added, and the reaction was allowed to proceed under mixing at 78 °C for 5 h [8,43]. Acetone was added to avoid a very rapid increase of the viscosity. Then, after allowing the temperature to drop to 60 °C, TEA (in equivalent ratio with respect to the acid groups of DMPA) was added, followed by 0.5 h stirring. Finally, the mixture was vigorously stirred (800 rpm) for 0.5 h, while distilled water (100 mL) was added into the reactor to produce the PU dispersion. The final dispersion was fed into a rotary evaporator to eliminate the remaining acetone (at 30 °C). The waterborne polyurethane was coded WBPU. 

### 2.3. Synthesis of Nanocelluloses

BC was obtained from *Komagataeibacter medellinensis* (previously named as *Gluconobacter medellinensis* sp.nov.), isolated from vinegar [44,45]. The bacteria were grown in a commercial H&S medium (2 wt./vol.% of glucose, 0.5 wt./vol.% of peptone, 0.5 wt./vol.% of yeast, 0.27 wt./vol.% of Na_2_HPO_4_, and 1.15 g/L of citric acid, at pH = 3.5). Incubation was carried out at 28 °C in an incubator oven for one week. The films were removed from the medium and to remove residues from the culture medium, and they were treated with 5 wt.% potassium hydroxide for 12 h at room temperature. Finally, the BC films were washed with distilled water until reaching a neutral pH value. 

It should be noticed that the strain used here produces a large amount of cellulose at low pH (3.5), indicating that it exhibits high tolerance to acidic environments while optimally producing BC. This is highly desirable in industrial fermentation processes, because microbial contamination can also be significantly reduced, since most microorganisms are unable to grow at low pH [45].

CNCs were synthetized using the acid hydrolysis of MCC, according to a previously reported technique used in our laboratory [46]. MCC was added in distilled water and dispersed by mechanical stirring until the suspension was homogenized. Then, concentrated sulfuric acid was slowly added, keeping the temperature below 20 °C with an ice bath. After that, the temperature was set to 44 °C, and the suspension was mixed with vigorous stirring for 2 h. Afterwards, the suspension was diluted with distilled water (*v*:*v*, 1:4) and then dialyzed against distilled water and bi-distilled water, until the water pH was reached. A polyester cloth with a pore size of 18 µm was used in order to filter the CNC suspension and separate potential remaining unreacted MCC.

### 2.4. Composites Preparation

A neat WBPU film was prepared by casting (30 °C overnight). In the case of composite films, two different paths were followed. 

The impregnation of a wet BC membrane by the WBPU dispersion was carried out to prepare the composite. The WBPU dispersion was added to the BC membrane placed in a glass Petri dish coated with a non-stick adhesive paper. Impregnation was carried out for a full day at room temperature, followed by drying the film in a convection oven at 30 °C overnight. A concentration of 1.35 wt.% of BC was achieved with this method.

**Figure 1:**
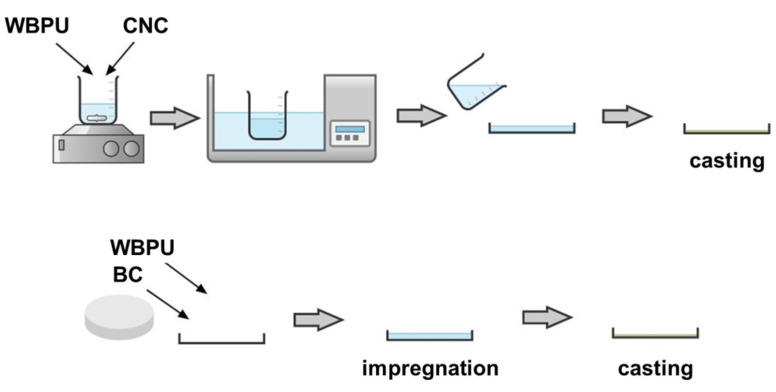
Preparation of the films containing waterborne polyurethane (WBPU) and cellulose nanocrystals (CNCs) or bacterial cellulose (BC).

A CNC composite film was prepared by mixing the two aqueous suspensions, the matrix (WBPU) and the reinforcement, with mechanical stirring for 30 min (750 rpm) and bath sonication for 5 min (37 Hz; 100% power; 5 min). The casting of the mixed dispersion in a glass Petri dish coated with a non-stick adhesive paper at 30 °C overnight was performed to achieve a concentration of 1.0 wt.% CNC (dry base). Figure 1 shows a simple scheme of both preparation processes.

### 2.5. Characterization Methods

A Bruker IFS 25 spectrometer at ambient temperature, with an attenuated total reflectance (ATR) unit, was used to obtain the FTIR spectra of the WBPU. The infrared spectra were recorded at 64 scans with a resolution of 4 cm^–1^. The composite films were also characterized with this technique. 

To determine the crystallinity of the cellulose and the influence as a reinforcement in the composites of the WBPU, an X-ray diffractometer (X PANalytical X’ Pert PRO, with Cu (Kα) radiation; wavelength: 1.54187 Å). Samples were scanned at 2θ from 5° to 60°, at a scanning speed of 0.016° s^−1^.

A differential scanning calorimeter (DSC Pyris 1 Perkin Elmer, with an electric intracooler as a refrigerator unit) was used in order to obtain the differential scanning calorimetry (DSC) thermograms of the WBPU and its composites. The samples were scanned from −70 °C to 200 °C at 10 °C min^–1^ under N_2_ atmosphere. 

The thermal stabilities of the WBPU and its composites were characterized using a TGA-50 Shimadzu. The samples were heated up from room temperature to 500 °C at a heating rate of 10 °C min^–1^ under N_2_ atmosphere.

A rheometer (Anton Paar Physica MCR 301) was used to determine the viscoelastic properties of the samples, by the dynamic torsion of solid rectangle bars in the range of linear viscoelastic behavior: dynamic mechanical analysis (DMA). The samples were tested from −80 °C to 140 °C at a scanning rate of 5 °C min^–1^ with a constant strain of 0.05% and a frequency of 1 Hz. 

The observations of the freeze-dried BC membrane and the cross-section surfaces of the films after cryogenic fracture were carried out using a scanning electron microscope (SEM) Jeol JM-6460LV with a voltage of 15 kV. The samples were previously placed in a sample holder and coated with gold and platinum. The CNC morphology was examined by field emission scanning electron microscopy (FE-SEM) using a Zeiss-Supra 40 microscope, with an accelerating voltage of 5 kV. The CNC dispersion was diluted at 0.001% and sonicated for 30 min; then a drop was put onto a holder to dry, followed by coating with a layer of gold. 

A goniometer OCA 15LHT Plus photo-microscope Dataphysics was used to measure the static contact angle of the composites and the neat WBPU films, using di-iodomethane (Sigma-Aldrich Corp.) and bi-distilled water at room temperature. Using a micropipette, a drop of 5 µL of each liquid was deposited on the surface of the samples. After 30 s (time to damp the drop oscillation), a photograph was taken using a high-resolution camera. Microsoft Photoeditor Software was used to measure the angle between the coating surface and the tangent line to the drop of liquid.

In order to register changes in the films, due to degradation/dissolution in water, the different samples were immersed in double-distilled water. Photographs were taken before immersion and after a specified test time. The samples were recovered from water using tweezers, and their surfaces were dried before the “after” pictures were taken.

## 3. Results and Discussion

### 3.1. Characterization of Nanocelluloses

#### 3.1.1. Microscopic Structure of the Nanocelluloses

**Figure 2:**
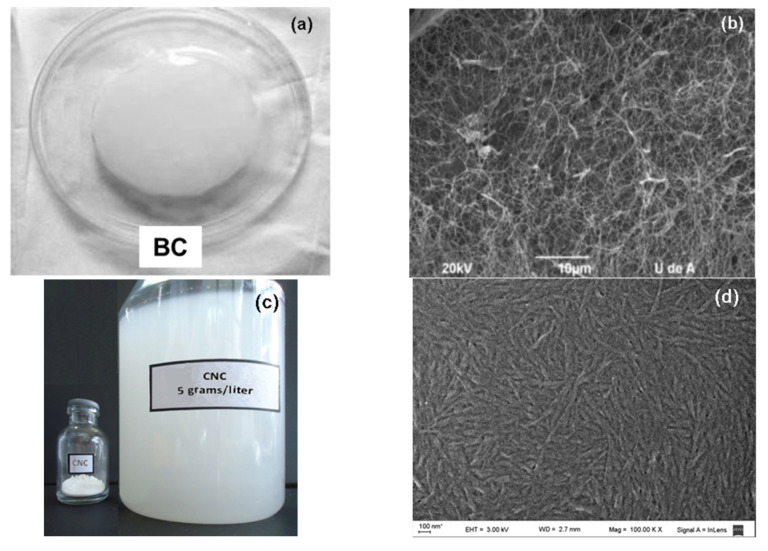
(**a**) Digital image of the wet BC membrane; (**b**) SEM image of the same sample after being lyophilized; (**c**) digital image of CNC powder and water dispersion; (**d**) field emission scanning electron microscopy (FE-SEM) image of the CNCs.

Figure 2a shows the typical gel-like appearance of a BC membrane, and Figure 2b shows the three-dimensional interconnected reticular pellicle formed by the nanosize ribbon-like fibers. The ribbons within the network were uniformly distributed and randomly oriented, probably because the microorganism duplicated the sites for cellulose synthesis before division, and therefore the mother and the daughters cells presented the same amount of active sites for synthesizing ribbons of constant dimensions. In this process, there was no break of the cellulose ribbon after splitting, only the creation of branch points [47,48].

Figure 2c,d shows the digital and FE-SEM images of the CNCs produced by the sulfuric acidolysis of the microcrystalline cellulose. The FE-SEM image allowed observing better the thin structure of the crystals: the size distribution was quite narrow and there were no traces of micrometer-sized fibers. 

The magnifications of the SEM images were selected to illustrate the main characteristics of the two nanocelluloses: the needle-like structure of the CNCs and the 3D entangled network of the BC, of which the fibrils were nanometric in thickness, but micrometric in length.

#### 3.1.2. FTIR Characterization

**Figure 3:**
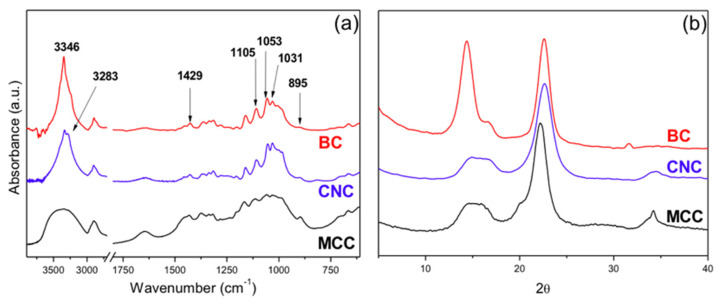
(**a**) FTIR spectra of the two nanocelluloses: BC and CNCs; (**b**) X-ray diffraction spectra of the BC and the CNCs.

Figure 3a shows the IR spectra of the two nanocelluloses used in the study. The characteristic peaks of cellulose type I were present in these spectra [49]. A peak corresponding to the symmetric bending of CH2 appears at 1429 cm^−1^ [50], a peak corresponding to the stretch of the C–O–C bond appears at 1105 cm^−1^, and a peak corresponding to the band appears at 895 cm^−1^ due to the glycosidic β-linkage of cellulose [51,52,53,54,55,56,57,58,59,60].

The comparison of the two spectra showed that the bands in the 1500–895 cm^−1^ region are of relative lower intensity in the CNC spectrum. According to previous publications [61], this suggests that the CNCs are less crystalline than BC, a difference that will be further considered in the analysis of the X-ray diffraction characterization.

There were also differences in the absorption band of the OH groups (3650–3120 cm^−1^), which were related to differences in the H-bonding present in the two nanocelluloses, with the free OH appearing at higher wavenumbers.

Another important difference between the spectra is the presence of the C–O–S absorption at 816 cm^−1^ in the spectrum of the CNCs, which is absent in the other cellulose samples. In previous publications, this peak is located in the 832–807 cm^−1^ range [62,63,64], and although some peaks were also expected in the region of 1200–1000 cm^−1^ [64], these were not spotted because C–O–C absorption occurs in the same region. Thus, the FTIR characterization further supported the presence of sulfate groups on the CNC surface due to the process used in its preparation.

#### 3.1.3. X-ray Diffraction Characterization

The X-ray diffractograms of the two nanocelluloses are shown in Figure 3b. Although both spectra showed that the samples corresponded to cellulose type I, the hydrogen bonding between and within cellulose molecules were different in the two celluloses used. Thus, BC was rich in cellulose type I_α_, while CNCs showed the typical spectrum of cellulose type I_β_ [65,66].

The peaks in the BC spectrum were assigned to the crystallographic planes (100), (010), and (110), corresponding to the 2θ = 14.4°, 16.7°, and 22.6°, respectively [44]. The high intensity of the peak corresponding to plane (100) is due to the strong monoplanar structure of the fibers of BC that had a ribbon-like structure and are preferentially oriented parallel to the surface of the film during drying [44,47,67]. This feature is characteristic of BC, although the relative height of the peaks varies with the substrate of the culture [68,69]. 

On the other hand, the X-ray diffraction spectrum of the CNCs showed the peaks corresponding to planes (101), ($10\bar{1}$), and (002) appearing at 2θ = 14.8°, 16.7°, and 22.6°, respectively. In this case, as it is typical from cellulose of high-order plants, the peak with the highest intensity corresponds to plane (002).

The calculation of the degree of crystallinity by a deconvolution method [70,71,72] led to the result that the BC was more crystalline that the CNCs and the original MCC, i.e., 80.79%, 71.43%, and 67.37%, respectively. The result is in agreement with the observation of the FTIR spectra as already discussed.

#### 3.1.4. Thermal Degradation (Thermogravimetric Analysis (TGA))

**Figure 4:**
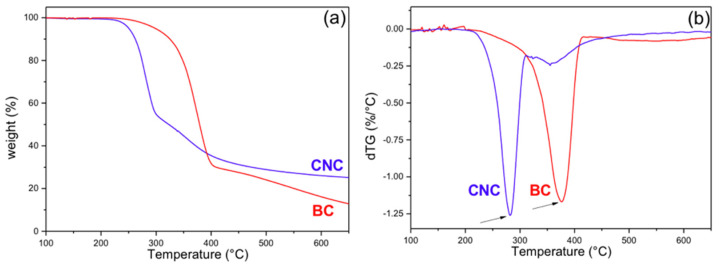
(**a**) Thermal degradation curves (thermogravimetric (TG) curves); (**b**) derivative signal (dTG) obtained under N_2_ atmosphere for the BC and the CNCs.

Figure 4 shows the TG and derivative signal resulting from the thermal degradation under N_2_ atmosphere of the two celluloses and after an initial loss of water. While the main degradation of BC occurred between 300 and 400 °C (Figure 4a) with the maximum peak at 375 °C (Figure 4b), in agreement with the cellulose degradation profile [73,74,75], the main degradation of the CNCs occurred in the range of 230–300 °C (Figure 4a) with a peak at ~281 °C (Figure 4b). This low-temperature degradation is the result of the preparation method, which left sulfate groups on the surface and reduced the thermal stability of the CNC. In addition, a minor degradation step centered about 350 °C appears. Furthermore, due to the presence of sulfate groups in the CNC sample, the final char was higher for this sample than for the BC one (24% and 13%, respectively, at 650 °C) [38,76,77].

Roman and Winter [38] found that even a small concentration of sulfate groups on the surface of nanocrystals obtained from BC is enough to produce a large reduction of the degradation temperature of the nanoparticles. The elimination of the sulfate groups requires relatively low energy and facilitates the depolymerization of the cellulose, beginning with the chains close to these groups [74,78]. The second minor peak that appears in the degradation of CNCs is due to the decomposition of the solid remains from the previous step. The sulfate groups are also responsible for the higher char in the CNC sample, and it has been reported that they also have a flame-retardant effect [38]. Our results are in agreement with those observations.

### 3.2. Characterization of the Composite Films

#### 3.2.1. Optical Aspect and SEM Topology

**Figure 5:**
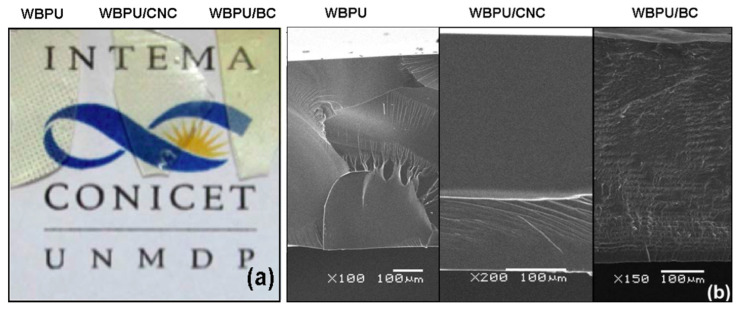
(**a**) Digital photography of the films; (**b**) SEM images of the fracture surface of the films. In both cases, the images correspond to neat WBPU (left), WBPU/CNC (center), and WBPU/BC (right) composites.

Figure 5a shows the images of the neat WBPU and the nanocomposite films. In the image of the neat WBPU film, it can be seen that it copied the texture of the Teflon film used as a base in the Petri dish. All films were optically transparent, at least in the range of thickness used in the study (600–650 µm).

The SEM images (Figure 5b) showed that low temperature fracture resulted in a brittle fracture with mirrorlike characteristics and river marks in some regions. The addition of 1 wt.% of CNCs did not qualitatively change the aspect of the fracture that was also brittle. However, the addition of 1.35 wt.% of BC resulted in a completely different topology. The image showed an arrangement of layers, which could be related to the growth of the BC as a network of fibrils that were added as new layers as the culture proceeded. Castro et al. [79] showed a similar layered topology for a nanocomposite made with PVA and obtained from a BC grown in the same strain that the one used in this work. On the other hand, the CNCs remained randomly dispersed in the WBPU, and the low concentration did not allow observing their presence.

#### 3.2.2. FTIR and DRX Analysis of Composites

**Figure 6:**
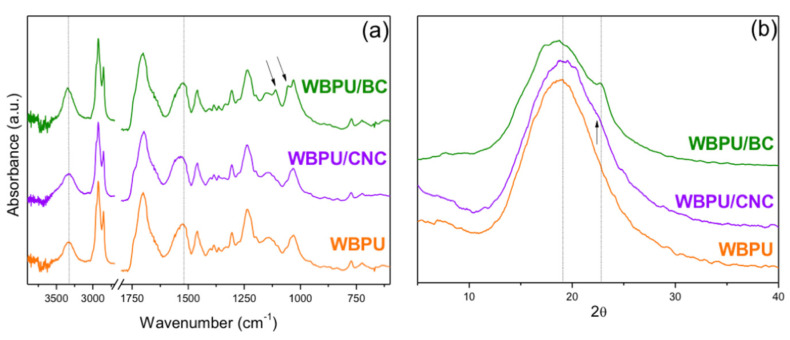
(**a**) FTIR spectra; (**b**) X-ray diffraction spectra of the neat WBPU and the nanocomposites.

Figure 6a shows the FTIR spectra of the films. The characteristic peaks of cellulose and WBPU appeared in the region of the bands located between 3500 and 3100 cm^−1^, overlapped in the composites and centered at 3335 cm^−1^, corresponding to OH groups and mainly to the –N–H absorption in the PU [19]. It can also be observed that the intensity of this peak was slightly higher for the BC composite than that of the CNC composite, which may be attributed to the fact that in the latter, the surface OH groups have been partially replaced by sulfate groups [38,76,77]. 

The absorbance at 1710 cm^−1^ is attributed to the hydrogen bonding of carbonyl stretching in amide I of the urethane bonds [80]; in the case of neat WBPU films, this peak appeared at 1702 cm^−1^ and was slightly shifted to 1697 cm^−1^ for the WBPU/CNC composite, which may be due to the hydrogen bonding developed between the polymer and the reinforcement [18]. Additionally, it was observed that the peak at 1527 cm^−1^ shifted to a longer wavelength of 1537 cm^−1^ in the case of the CNC composite. This peak is attributed to the N–H bending vibration of the urethane group of the WBPU (amide II). Thus, the shift supports the already mentioned interactions between this reinforcement and the WBPU [81,82]. However, no shifts were observed in any of these two regions (1702 and 1527 cm^−1^) in the spectrum of the BC composite. Finally, in the case of the composite with BC, the peaks marked with arrows in the zone between 1500 and 899 cm^−1^ corresponded to the absorption peaks of BC.

It is probable that the large specific surface area of the CNCs, containing hydroxyls and sulfate groups, leads to more H-bonding interactions with the PU. These types of interactions have been previously reported for a CNC composite made from a biomacrodiol and 1,6 hexamethylene diisocyanate (HDI). The authors showed that an increasing content of CNC produces an increase of the C=O bond absorption at least up to the CNC percolation [18]. Minor changes were also reported for a similar composite in the ~1530 cm^−1^ region [83]. This report is in agreement with the shifts in the amide I and amide II regions observed in the present work. 

Figure 6b shows the X-ray diffraction spectra of the neat polymer and the nanocomposites. The amorphous nature of the WBPU resulted in a wide peak observed in the three spectra. However, the addition of BC can be confirmed by the presence of an overlapping small peak at ~22.6°, corresponding to plane (110) of the BC. On the other hand, the addition of CNCs only produced a very small shoulder in that same 2θ region (corresponding to plane (002) of the CNCs). Additionally, a small shift of the amorphous peak towards higher angles can be detected. This change may be related to the good dispersion of the CNCs and the interactions with the polymer structure, which were discussed in the FTIR section.

#### 3.2.3. Thermal Characterization of the Films (DSC, DMA, and TGA)

**Figure 7:**
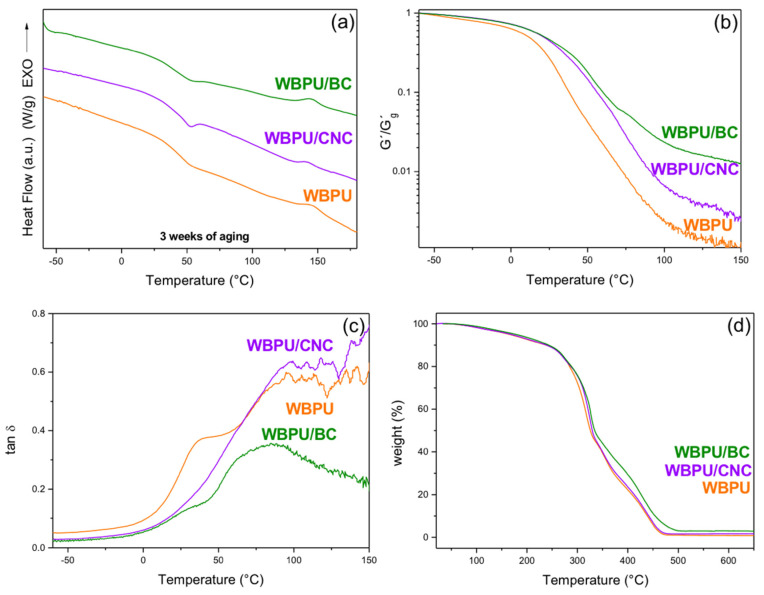
(**a**) Differential scanning calorimetry (DSC) traces with 3 weeks of aging; (**b**) normalized storage modulus; (**c**) tanδ of the films; (**d**) TG signal (residual weight %) of the films of the neat WBPU and the nanocomposites.

The DSC characterization showed that a thermal event occurred at 56.4, 56.7, and 57.8 °C for the WBPU, CNC composite, and BC composite, respectively, which is associated to the glass transition temperature of the materials (Figure 7a). An endothermic peak associated to the event, which is due to densification of the PU during storage, appeared in the DSC curves of the composites [84]. In those cases, to characterize the aged samples, the peak of the endotherm was considered as an estimation of the T_g_ reached in those conditions. After three weeks from preparation, densification took place in the composites; however, the endotherm was not present in the curve of the unreinforced WBPU. This suggested that the presence of the nanocelluloses accelerated the densification of the material.

Figure 7a also shows an exothermal event occurring around 150 °C, which is associated to a short-range order–disorder transition corresponding to the hard segments of the WBPU (region associated to the reacted isocyanate molecules) [82,85,86,87].

The dynamic mechanical analysis (Figure 7b) showed that the thermal transition from glass to rubber occurs in a wide temperature range from about room temperature to above 100 °C. The curves of the normalized storage modulus (G’/G’_g_) allowed clearly observing the effect of the nanoparticles, producing a shift of the T_g_ of the composites to higher temperatures. The tanδ plot (Figure 7c) showed that the neat polymer had actually two relaxations, with one close to room temperature and other one being more intense at around 90 °C. In the curves of the nanocomposites, the low temperature relaxation was much reduced by the presence of the nanoparticles and relatively more in the case of the CNC films. On the other hand, the reduction of the main relaxation at higher temperatures was more obvious in the case of the BC, indicating that the mobility of all the polymer network structure was reduced. Similar results have been reported by other authors for cellulose reinforced composite materials [88].

It is also interesting to compare the storage moduli of the different samples in the rubbery region. Clearly, the addition of the nanocelluloses resulted in the increase of the rubbery modulus, which was expected because of the high modulus of the cellulose (~20–100 GPa) [23,89,90], compared to that of the rubber modulus of the WBPU (G’ at 100 °C was 1.3 MPa). The interfacial interactions (already discussed) led to strong interfacial adhesion and good stress transfer from the matrix to the nanocelluloses, which resulted in effective reinforcement of the polymer [18,91,92]. The comparison also showed an additional interesting feature: the reinforcement of the BC was higher than that obtained with the CNCs. For example, at 100 °C, the addition of the CNCs resulted in a 4 times increase of the normalized modulus relative to the neat WBPU, but more than 10 times increase in the BC case. This higher reinforcement is the result of the 3D cellulose network, which also led to the reduction of the tanδ peaks as it was already discussed. 

Figure 7d shows the thermal degradation traces of the neat polymer and the nanocomposites, where only the BC composite showed a small improvement in the thermal degradation. The temperature at which the 5% weight loss occurred was shifted from 167 °C to 168 °C and 178 °C for the WBPU/CNC and WBPU/BC composites, respectively. The same trend was observed in the temperature, at which 90% of the weight was lost from 438 °C for the neat WBPU to 441 °C and 454 °C for the nanocomposites containing CNCs and BC, respectively. All samples showed a very low residual char, although slightly higher in the case of the composites compared to that of the neat WBPU. 

As it is typical in PUs, the thermal degradation initiates with the breakage of the urethane bonds, then the decomposition of the polyol and finally C–C cleavage take place. In the present case, the addition of the nanocelluloses had little effect on the thermal degradation of the polymer, although a minor improvement was seen for the CNC nanocomposite (in the 280–310 °C range) and above 280 °C for the BC nanocomposite. A similar behavior was reported for a CNC/WBPU composite made from a biomacrodiol and HDI. It was found that increasing contents of CNC produced increased stability in a similar temperature region. The authors explained the improved thermal stabilization by the interactions between the CNCs and the urethane groups of the polymer [18,91,92]. The slightly better effect of adding BC may be explained as the result of the network formed by this nanocellulose, which as it is degraded can form a char layer that acts as a retardant or barrier protecting the polymer [92]. Zhang et al. made an analogous finding working with CNCs and CNFs as reinforcements of polyhydroxybutyrate. They found that CNFs, which also form an entangled network (similar to BC, but different from CNCs) lead to a nanocomposite of higher thermal stability than the one prepared with CNCs. They explained their result due to the reticular structure formed by CNFs at the micro level that reduces the degradation rate of the polymer [93].

#### 3.2.4. Static Contact Angle and Water Absorption

**Figure 8:**
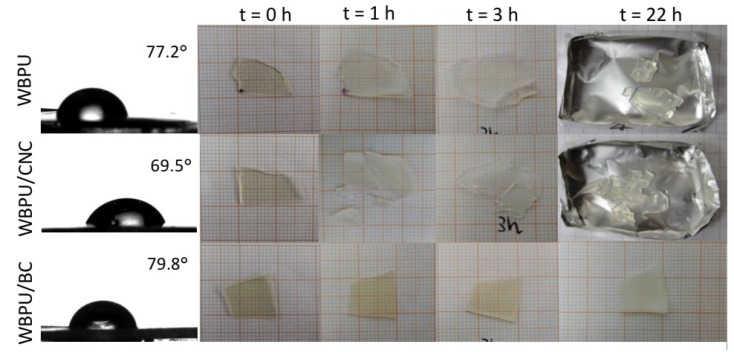
Behavior of the films in contact with water: contact angle and water immersion effects on the integrity of the films.

Figure 8 presents a summary of the results on static contact angle of water on the surface of the films. The upper surface of the films was used for the measurements. In all cases, particularly in the case of the CNC nanocomposite, the angle was lower than 90° (hydrophilic surfaces), showing little change in the case of the WBPU/BC composite with respect to the unfilled polymer. Considering that nanocelluloses are hydrophilic and that BC has been identified as more hydrophilic than CNCs [94], the explanation for these results could be found in the processing and distribution of the celluloses in the films. CNCs were randomly distributed through the whole sample, since the distribution was obtained via mixing and ultrasonic stirring. Instead, the BC composite films were obtained by the immersion/impregnation of the wet BC membrane. The last procedure is more prone to lead to the formation of a thin polymer region on the surfaces of the film than the procedure used with CNCs. Figure 5b also supports this point of view, since the layers of BC that can be seen in the internal part of the cross section of the film were not present close to the surfaces Thus, CNCs would be more exposed than the BC network, and thus, surface hydrophobicity would be higher in the WBPU/BC composite than in the WBPU/CNC composite.

Additionally, the films were immersed in bi-distilled water for different lengths of time (Figure 8), and the result was the decohesion of the materials that broke into small pieces, with the exception of the BC nanocomposite. As it has been previously discussed, the BC forms a network of ribbons producing an interpenetrated network with the WBPU. The BC network is responsible for restricting the swelling and ultimately the fragmentation of the film.

## 4. Conclusions

Optically transparent bio-composite films from CO-based WBPU reinforced with CNCs and BC were obtained by means of two simple processing methodologies. The hydrophilic characteristics of both reinforcements favor its dispersion or impregnation with the WBPU aqueous dispersion.

The H-bonding between CNC and urethane groups of the WBPU was confirmed by the shift of the FTIR peaks corresponding to the urethane absorptions (amide I and amide II) in the spectrum of the nanocomposite. 

The thermal stability of the BC was higher than that of the CNC, since the preparation method (acid hydrolysis) of the latter left sulfate groups on the surface and reduced its thermal stability. Despite this, both bio-reinforcement slightly increased the thermal stability of the bio-composites with respect to the polymeric matrix, with BC improving the response at temperatures above 300 °C.

While the storage modulus of the WBPU was the same as that of an elastomer at 100 °C (1.3 MPa), both nanocelluloses had an effective reinforcing effect. The normalized storage modulus at that same temperature increased with respect to the neat polymer between 10 and 4 times for BC and CNC bio-composites, respectively. These results were a consequence of the interactions of the bio-reinforcement with the polymeric matrix. Particularly in the case of the BC-based biocomposite, the results would be associated to the three-dimensional interconnected morphology of the bio-reinforcement. Even more interesting, the tridimensional structure of BC allowed maintaining the structural integrity of the composites films when being immersed in water.

Water contact angle was affected, even at the low nanocellulose contents used in this work, with the CNC nanocomposite displaying the most hydrophilic surface (77.2° and 69.5° for the neat WBPU and the WBPU/CNC nanocomposites, respectively).

Overall, the preparation process was simpler and more controllable in the case of the CNC nanocomposite, with dispersions blends easily obtained at the required concentration and films produced by casting and drying. These characteristics would make it a better choice for coating applications. On the other hand, the structural cohesion of BC nanocomposites may be more appealing in the preparation of parts where the flexibility of the BC 3D network is a clear asset.

## Data Availability

The data presented in this study are available on request from the corresponding author.
